# Supplementary material for: Deposition of Lead Phosphate by Lead-Tolerant Bacteria Isolated from Fresh Water near an Abandoned Mine
Source: Int J Mol Sci. 2022 Feb 24;23(5):2483. doi: 10.3390/ijms23052483 (PMC8910126; doi:10.3390/ijms23052483)
Supplement: Supplementary file 1 [file ijms-23-02483-s001.zip › ijms-1594131-supplementary.pdf]

Supplementary materials

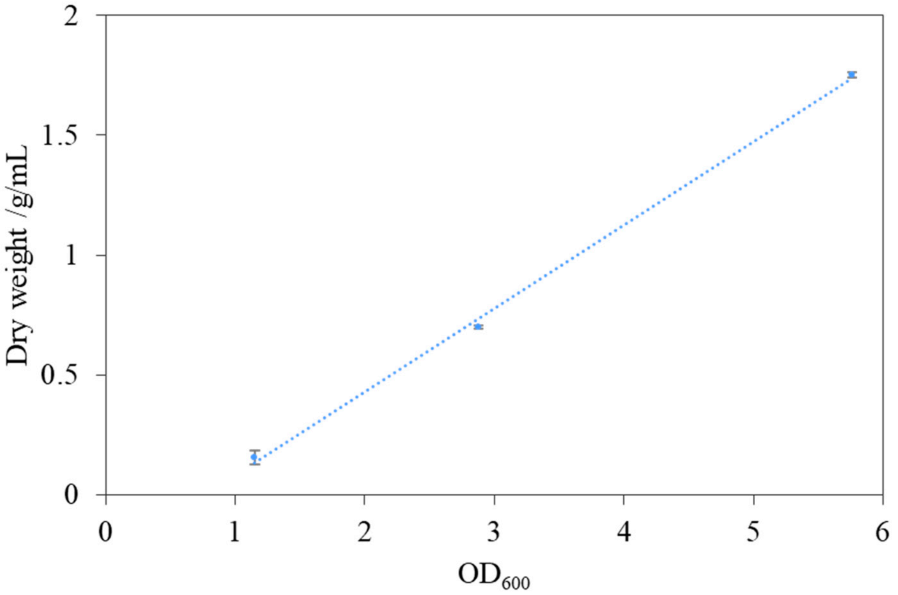

Figure S1. The correlation between dry weight of cells and OD_{600}.

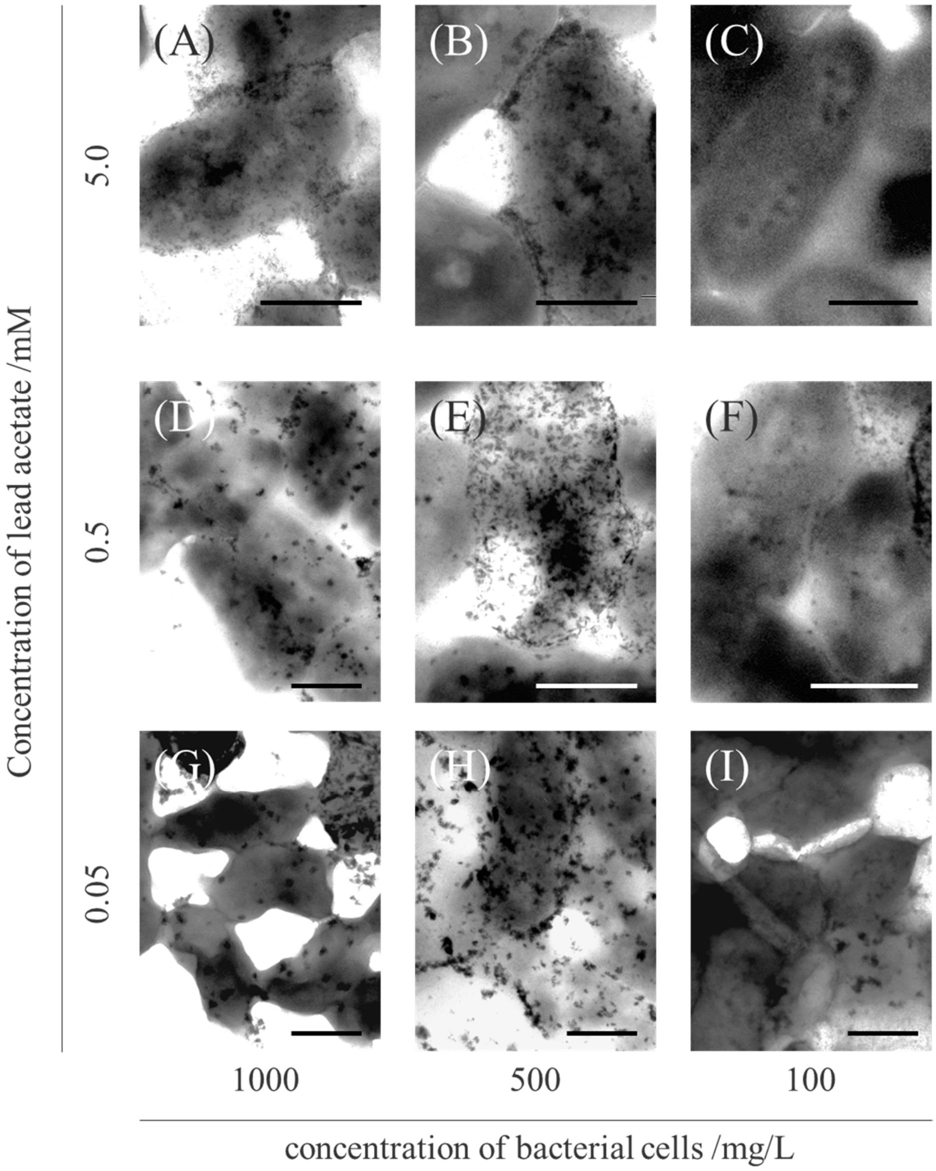

Figure S2. TEM images of KKY-29 incubated in various concentration of lead acetate solution. Scale bar is 500 nm.

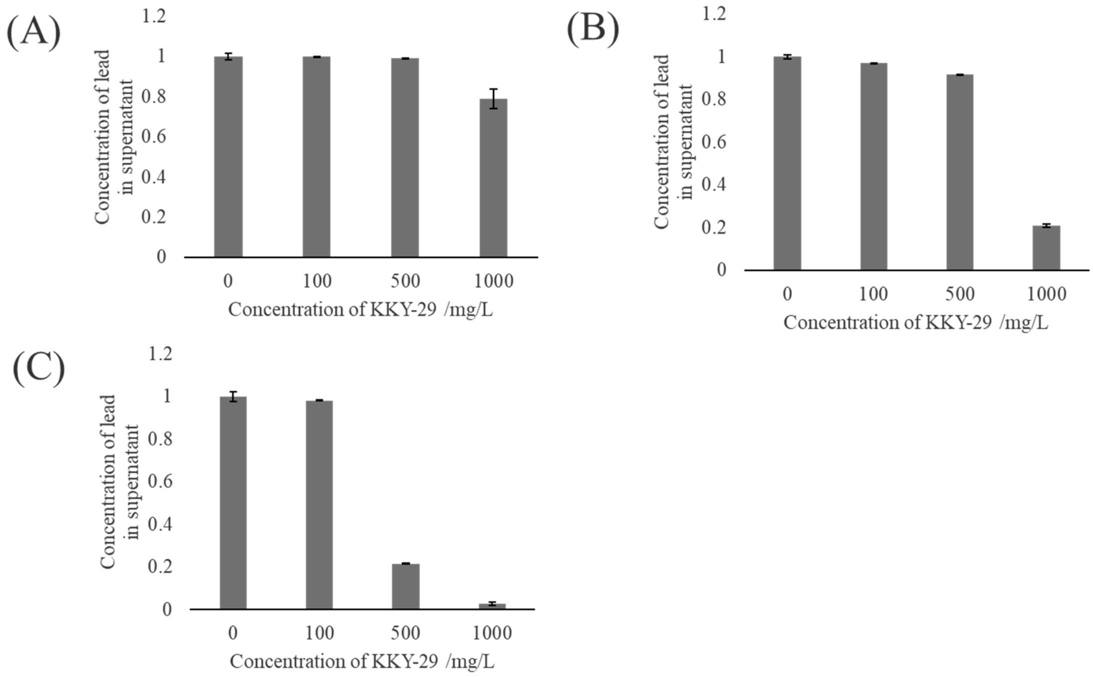

Figure S3. The lead concentrations of the KKY-29 treated with lead acetate solution ((**A**) 5.0 mM, (**B**) 0.5 mM, (**C**) 0.05 mM). The relative values are shown when the condition of no metal solution is set to 1 (n = 3, mean SD).

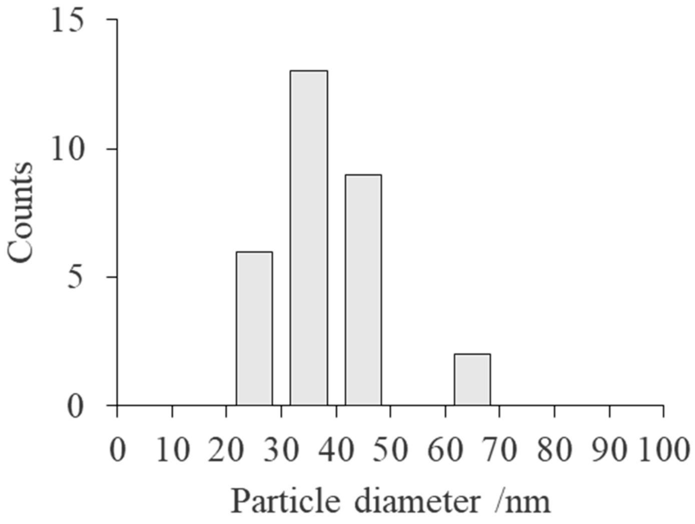

Figure S4. The size distribution of nanoparticles.

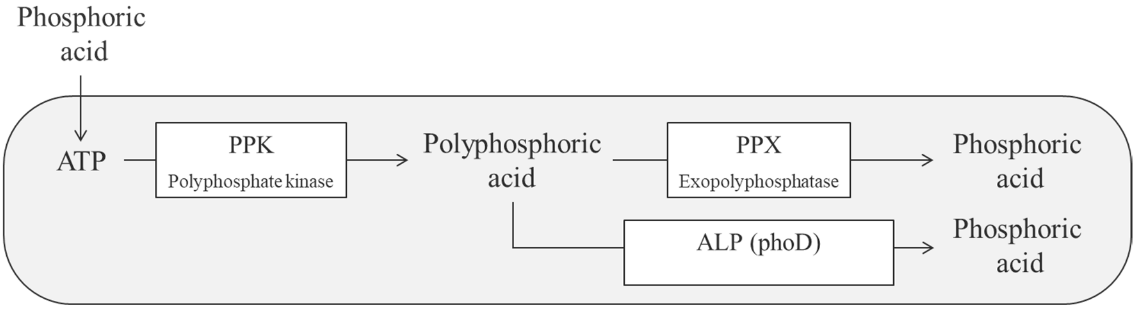

Figure S5. Schematic model of polyphosphoric acid decomposition pathway.

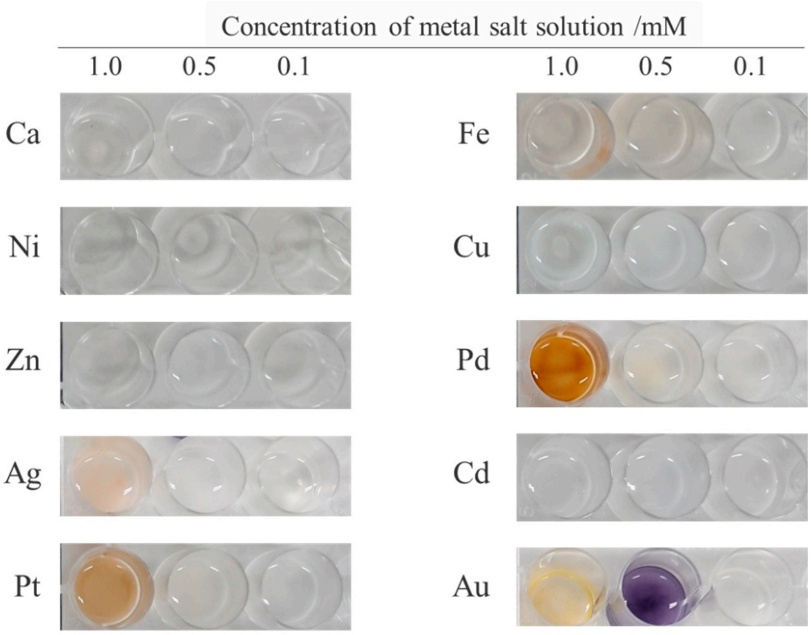

Figure S6. The color results of the KKY-29 suspension after incubating with various metal salt solution.

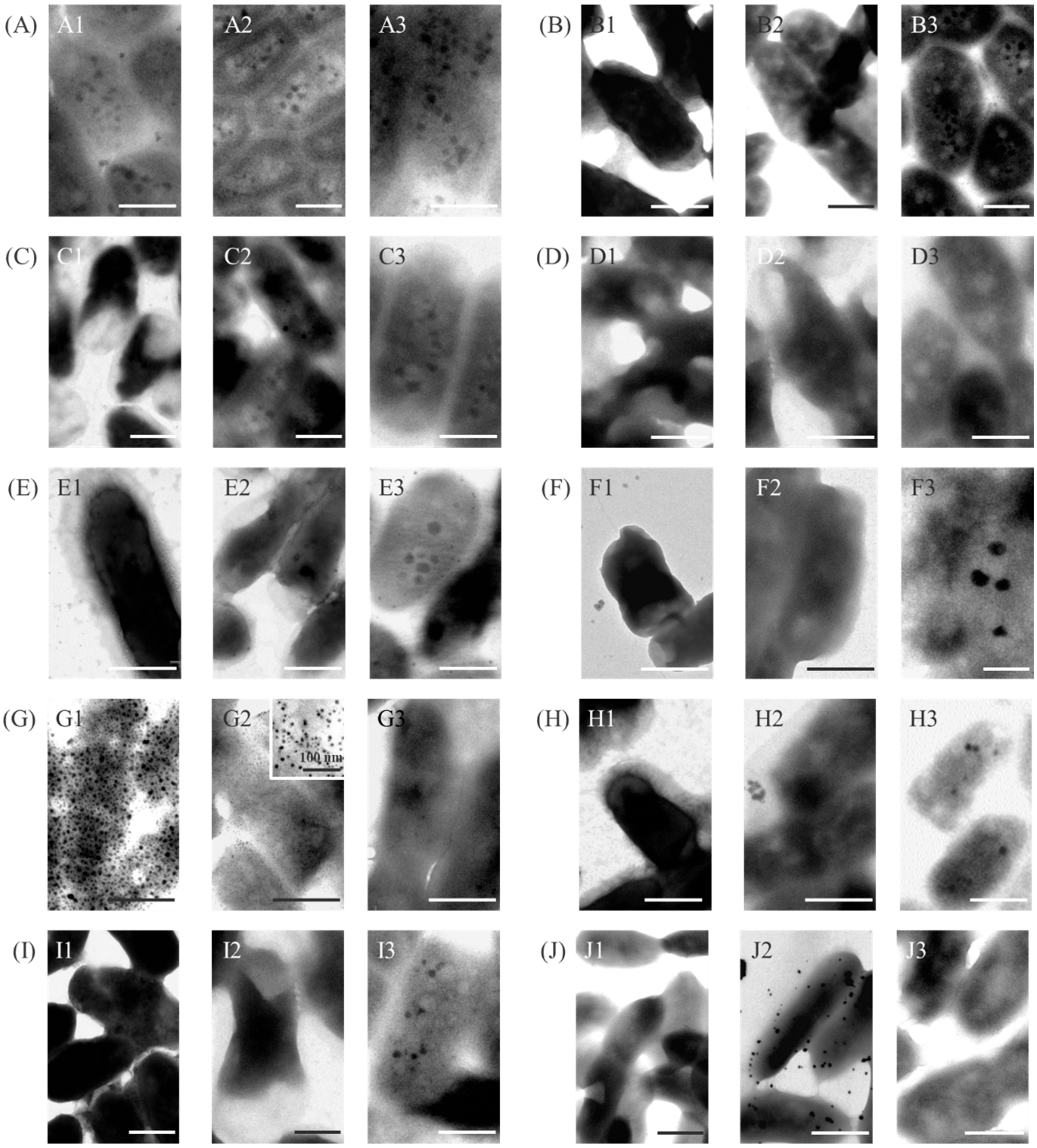

Figure S7. TEM images of the KKY-29 after incubating with various metal salt solution. Scale bar is 500 nm.

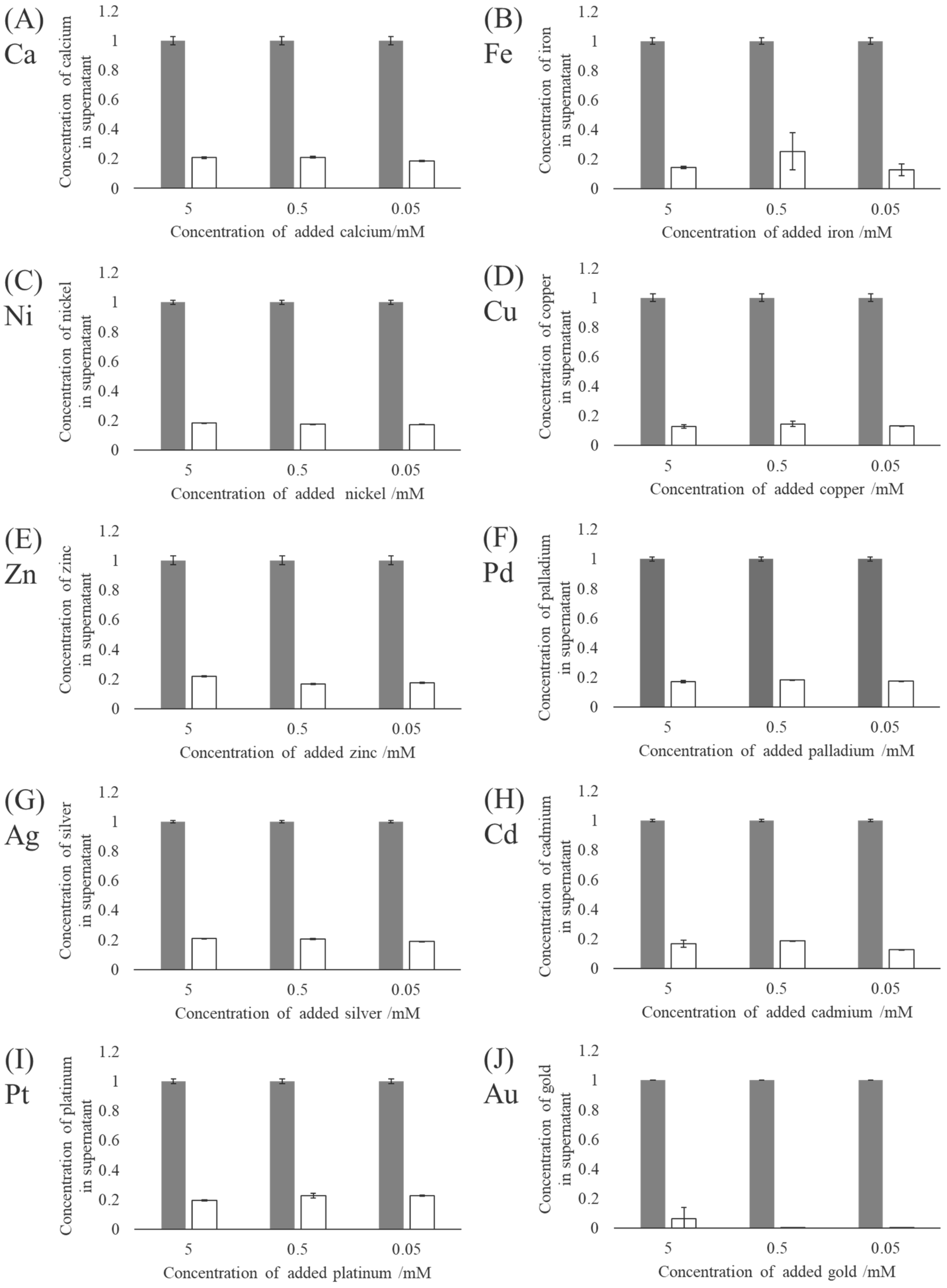

Figure S8. The metal concentrations of the KKY-29 treated with various metal solution (*n* = 3, mean SD). Closed bar means as negative control and open bar means samples with KKY-29.
